# Supplementary material for: Inertial Sensor-to-Segment Calibration for Accurate 3D Joint Angle Calculation for Use in OpenSim
Source: Sensors (Basel). 2022 Apr 24;22(9):3259. doi: 10.3390/s22093259 (PMC9104520; doi:10.3390/s22093259)
Supplement: Supplementary file 1 [file sensors-22-03259-s001.zip › sensors-1659724-supplementary.pdf]

Supplementary materials:

Markers and IMUs placement.

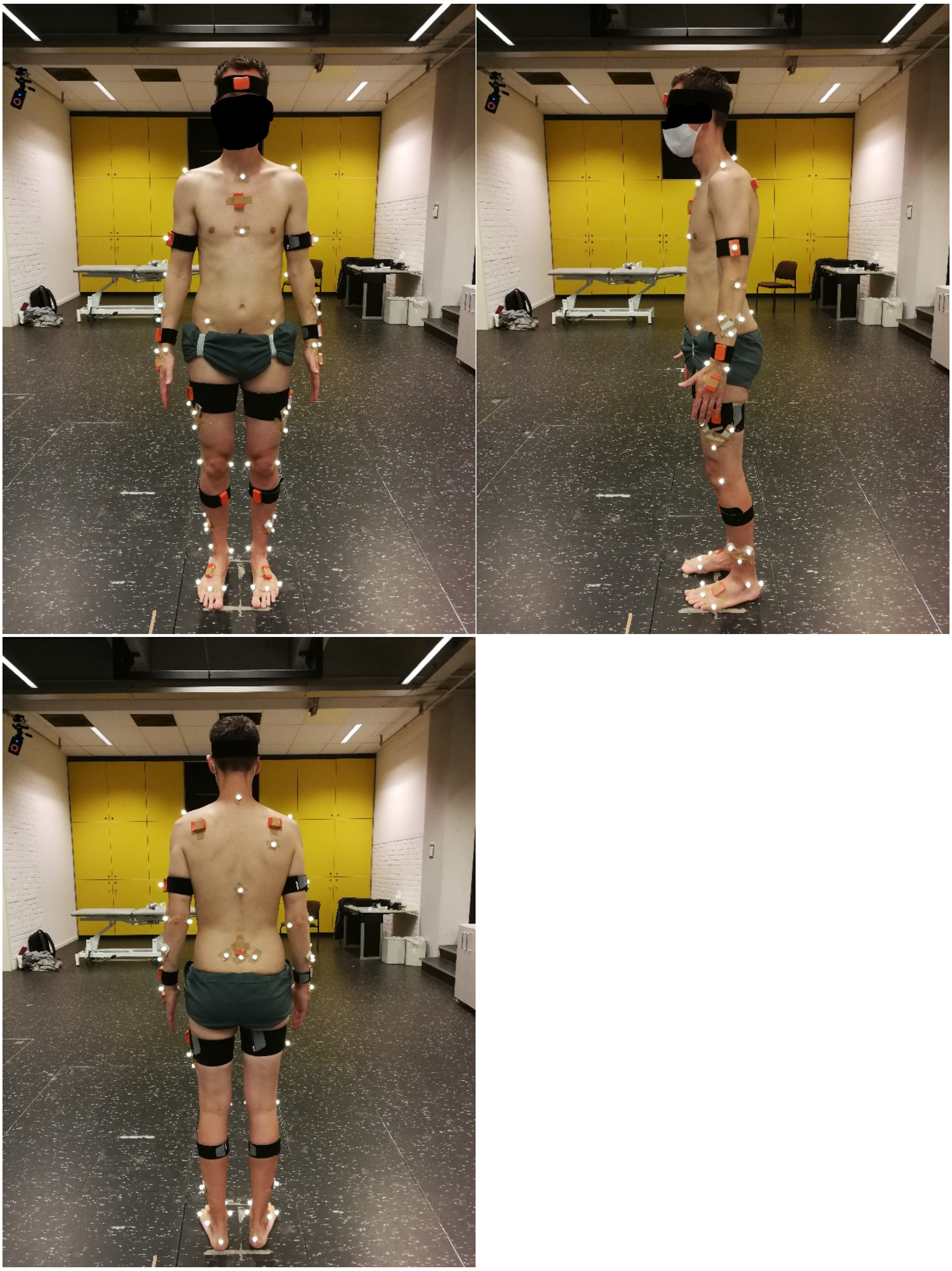

Figure S1: Markers placement (white bright points) and IMU sensors placement (orange boxes) with straps and tapes.

Table S1: Marker and sensor names and positions

MARKERS NAME	POSITION
C7	7th cervical
CLAV	clavicle - in between in the centre
STRN	sternum - at the end in the centre
T10	10th thoracic
RBAK	right back - on the right scapula
LSHO	Left shoulder - at the acromio-clavicular joint
LUPA1	Left upper arm - between shoulder and elbow
LELB	Left elbow - lateral epicondyle
LFRA1	left forearm - between the elbow and wrist
LWRA	lef wrist A - medial left wrist
LWRB	left wrist B - lateral left wrist
LFIN	Left finger - second metacarpal head
RSHO	right shoulder - at the acromio-clavicular joint
RUPA1	right upper arm - between shoulder and elbow
RELB	right elbow - lateral epicondyle
RFRA1	right forearm - between the elbow and wrist
RWRA	right wrist A - medial right wrist
RWRB	right wrist B - lateral right wrist
RFIN	right fing - second metacarpal head
LASI	right anterior superior iliac spine
RASI	left anterior superior iliac spine
RPSI	right posterior superior iliac spine
LPSI	left posterior superior iliac spine
LTHI	left thigh cluster
LTHI2	left thigh cluster
LTH3	left thigh cluster
LKNE	left knee - lateral femoral epicondyle
LKNEmed	left knee medial - medial femoral epicondyle
LTIB	left tibia cluster
LTIB2	left tibia cluster
LTIB3	left tibia cluster
LANK	left ankle - lateral malleolus
LANKmed	left ankle - medial malleolus
LHEE	left hee - on the calcaneous at the same height as the left foot second metatarsal head
LTOE	left toe - second metatarsal head
LLatFoot	left lateral foot

RTHI	right thigh cluster
RTHI2	right thigh cluster
RTH3	right thigh cluster
RKNE	right knee - lateral femoral epicondyle
RKNEmed	right knee medial - medial femoral epicondyle
RTIB	right tibia cluster
RTIB2	right tibia cluster
RTIB3	right tibia cluster
RANK	right ankle - lateral malleolus
RANKmed	right ankle - medial malleolus
RHEE	right hee - on the calcaneous at the same height as the right foot second metatarsal head
RTOE	right toe - second metatarsal head
RLatFoot	right lateral foot

IMU position

Torso	HEAD	On the forehead
	STERNUM	On the sternum
	PELVIS	On the sacrum
Upper Limbs	SHOU	On the scapula
	UARM	On the humerus
	FARM	On the radio
	HAND	On the back of the hand
Lower Limbs	LLEG	On the lateral part of the thigh
	ULEG	On the upper medial shank part
	FOOT	On the metatarsal

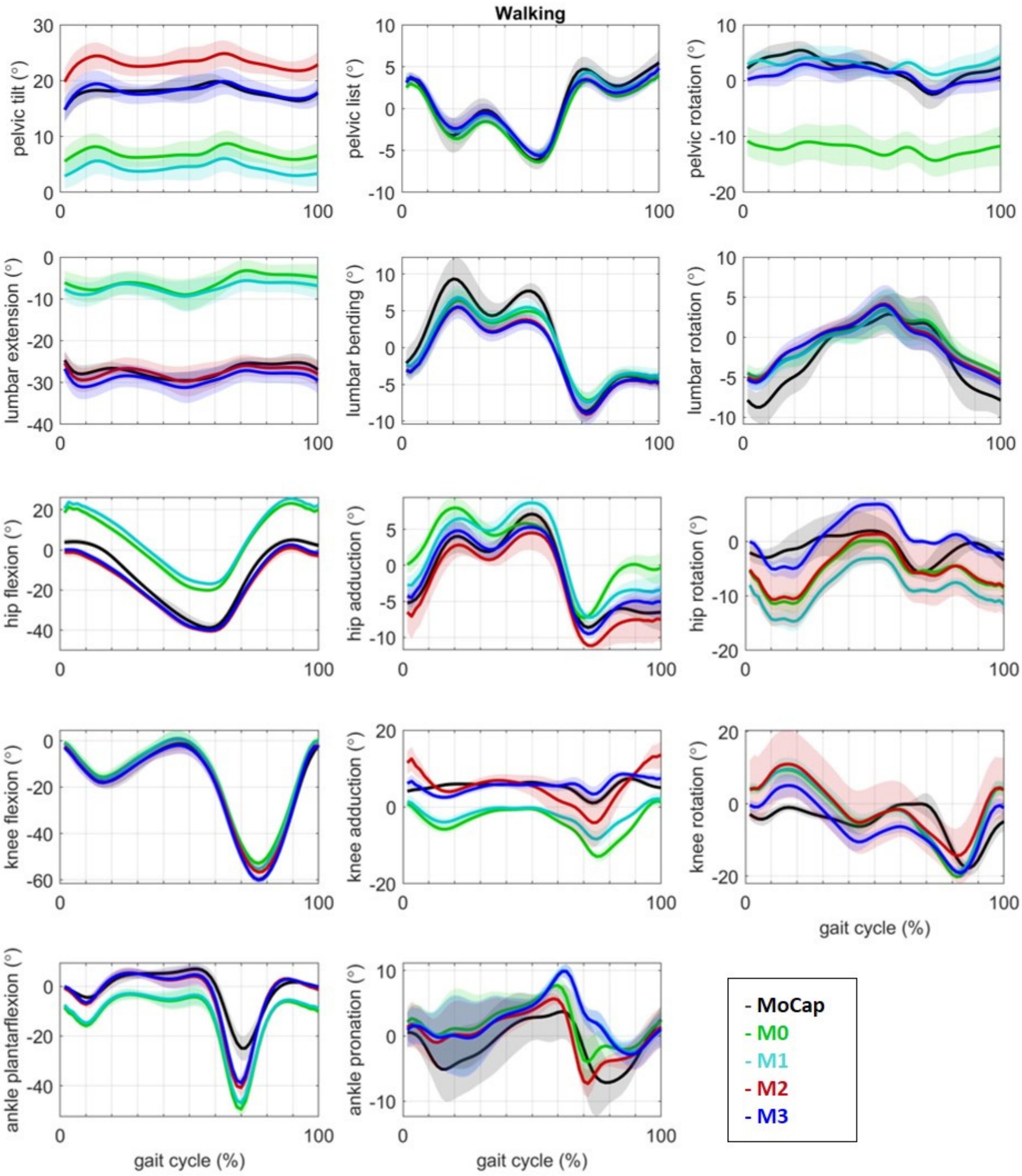

Figure S2: 3D pelvic, lumbar and lower limb joint angles during an over ground walking - gait cycle - with the InCap system over a cycle for a representative subject. InCap system (M0 - green, M1 - cyan, M2 - red, M3 - blue) and MoCap system (black). M0: OpenSense standard pipeline, M1: functional hip abd-adduction motion calibration, M2: functional PCA walking calibration, M3: functional hip abd-adduction and walking PCA calibration

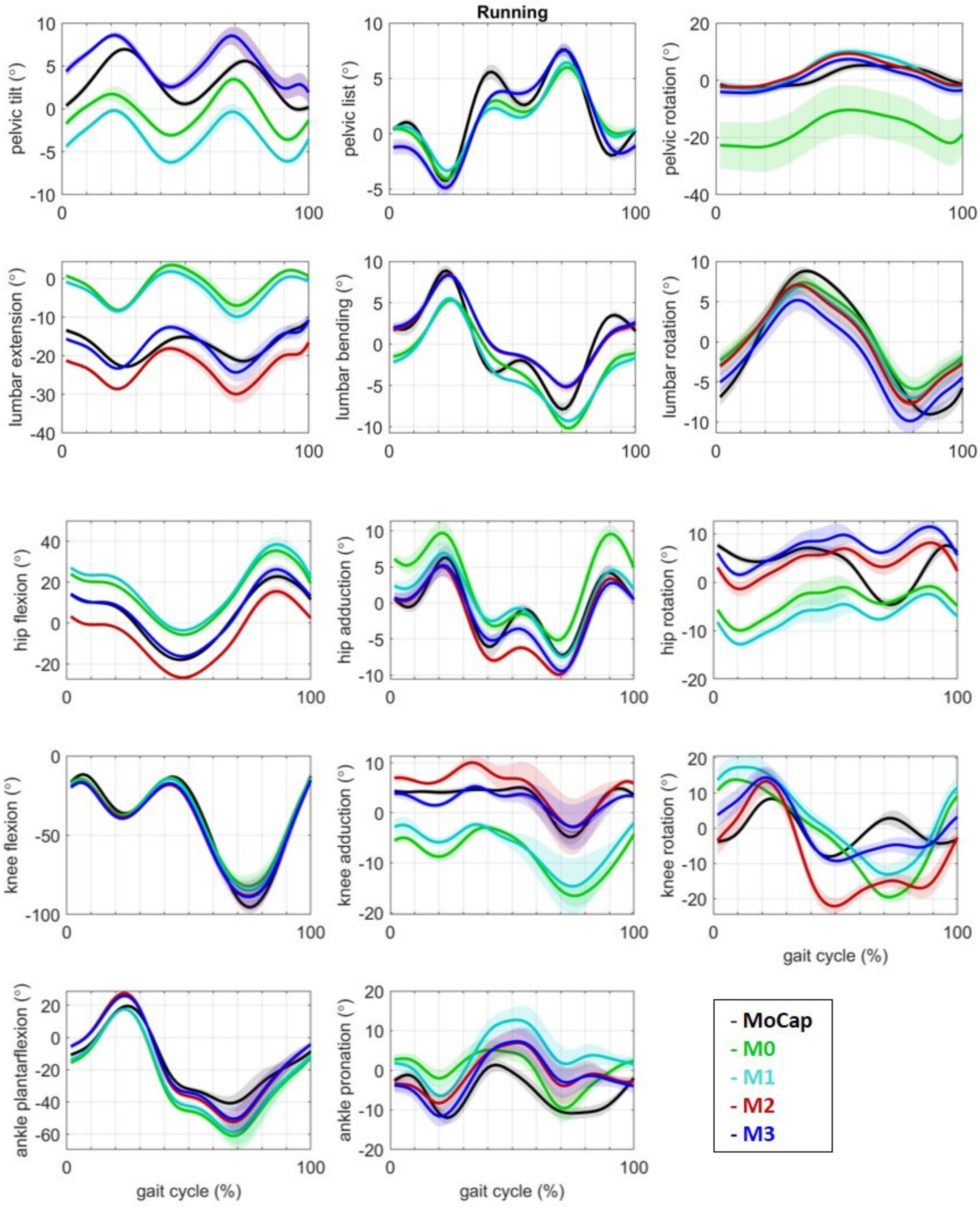

Figure S3: 3D pelvic, lumbar and lower limb joint angles during running - gait cycle - with the InCap system over a cycle for a representative subject. InCap system (M0 - green, M1 - cyan, M2 - red, M3 - blue) and MoCap system (black). M0: OpenSense standard pipeline, M1: functional hip abd-adduction motion calibration, M2: functional PCA walking calibration, M3: functional hip abd-adduction and walking PCA calibration.

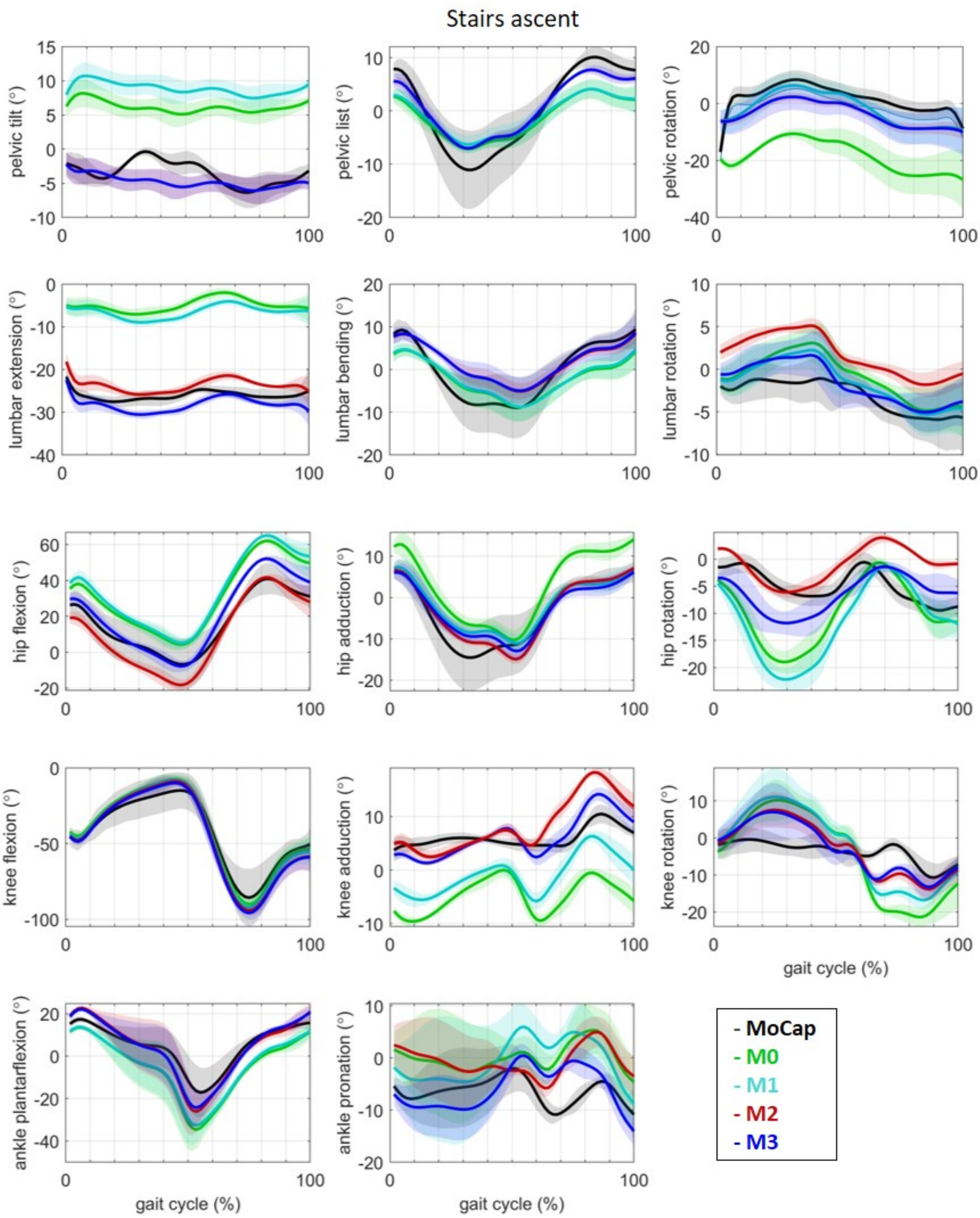

Figure S4: 3D pelvic, lumbar and lower limb joint angles during stairs ascent - gait cycle - with the InCap system over a cycle for a representative subject. InCap system (M0 - green, M1 - cyan, M2 - red, M3 - blue) and MoCap system (black). M0: OpenSense standard pipeline, M1: functional hip abd-adduction motion calibration, M2: functional PCA walking calibration, M3: functional hip abd-adduction and walking PCA calibration.

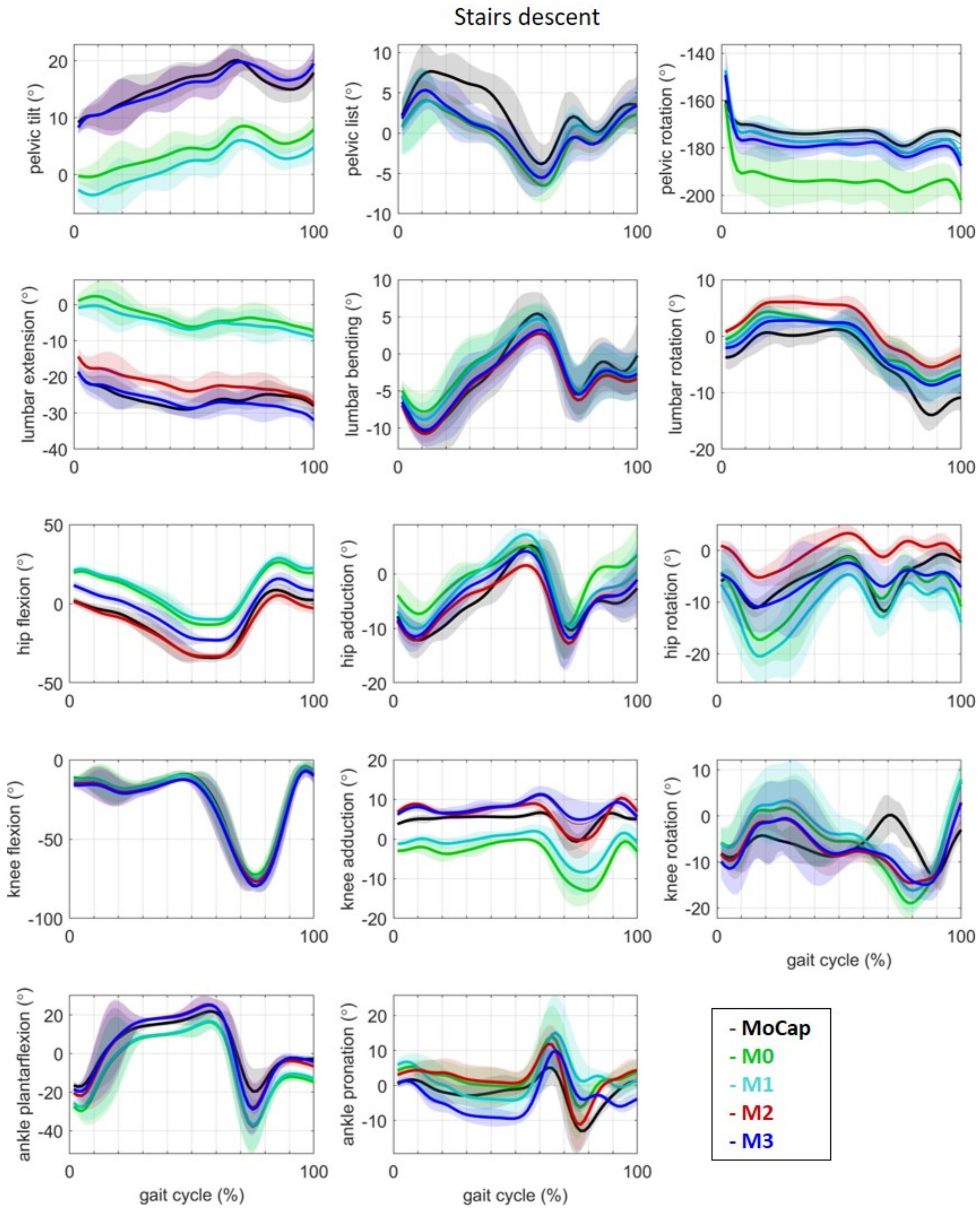

Figure S5: 3D pelvic, lumbar and lower limb joint angles during stairs descent - gait cycle - with the InCap system over a cycle for a representative subject. InCap system (M0 - green, M1 - cyan, M2 - red, M3 - blue) and MoCap system (black). M0: OpenSense standard pipeline, M1: functional hip abd-adduction motion calibration, M2: functional PCA walking calibration, M3: functional hip abd-adduction and walking PCA calibration.

Walking		RMSE(°)				R^2				ΔROM(°)				ΔPeak(°)			
		Method 0	Method 1	Method 2	Method 3	Method 0	Method 1	Method 2	Method 3	Method 0	Method 1	Method 2	Method 3	Method 0	Method 1	Method 2	Method 3
		Mean (SD)				Mean (SD)				Mean (SD)				Mean (SD)			
Pelvic	Tilt	5.4 (1.8)	5.7 (2.1)	4.4 (2.6)	4.4 (1.0)	0.5 (0.3)	0.5 (0.3)	0.5 (0.3)	0.6 (0.3)	1.2 (2.0)	1.3 (2.0)	1.3 (1.5)	1.3 (1.5)	5.3 (4.1)	5.9 (4.6)	2.0 (2.6)	2.6 (2.0)
	List	3.0 (1.1)	2.4 (0.8)	3.6 (0.9)	2.1 (0.8)	0.6 (0.3)	0.7 (0.3)	0.6 (0.3)	0.6 (0.3)	3.3 (1.4)	2.9 (1.4)	3.2 (1.4)	3.2 (1.7)	2.1 (1.9)	1.5 (1.6)	1.5 (2.2)	1.6 (1.6)
	Rotation	23 (8.2)	16 (9.1)	4.6 (2.0)	5.3 (2.1)	0.6 (0.3)	0.6 (0.3)	0.7 (0.3)	0.7 (0.3)	2.8 (1.9)	2.7 (1.9)	2.8 (1.7)	2.8 (1.7)	2.7 (2.4)	2.5 (3.2)	2.2 (2.5)	2.2 (2.4)
Lumbar	Extension	8.4 (3.2)	10 (4.4)	4.4 (4.4)	3.2 (2.8)	0.8 (0.3)	0.7 (0.3)	0.7 (0.3)	0.7 (0.3)	2.1 (2.1)	1.6 (2.3)	1.5 (2.1)	1.4 (2.2)	4.9 (5.5)	4.0 (4.6)	3.7 (3.5)	3.4 (3.2)
	Bending	4.2 (0.9)	3.0 (0.8)	2.7 (0.9)	3.5 (0.9)	0.7 (0.3)	0.7 (0.3)	0.8 (0.3)	0.8 (0.3)	3.2 (5.8)	2.8 (5.9)	2.9 (4.5)	2.9 (3.9)	3.2 (2.7)	2.3 (3.3)	3.7 (3.9)	3.1 (2.7)
	Rotation	4.2 (0.9)	4.5 (1.4)	5.7 (0.8)	3.3 (0.9)	0.9 (0.2)	0.8 (0.2)	0.9 (0.2)	0.9 (0.2)	2.7 (2.4)	2.5 (2.3)	3.0 (2.3)	2.9 (2.1)	2.2 (2.4)	3.6 (3.2)	2.2 (2.5)	2.2 (2.4)
Hip right	Flexion/Extension	6.7 (3.6)	5.4 (3.6)	3.2 (2.3)	2.9 (2.3)	0.9 (0.1)	0.9 (0.1)	0.9 (0.1)	0.9 (0.1)	3.2 (1.2)	1.8 (3.9)	1.7 (4.0)	1.7 (3.5)	4.5 (3.5)	5.4 (4.5)	5.2 (2.7)	4.2 (1.1)
	Abd/Adduction	**5.9 (1.9)**	4.2 (2.2)	5.9 (0.6)	2.4 (0.9)	**0.8 (0.2)**	0.9 (0.1)	0.7 (0.2)	0.8 (0.1)	1.6 (6.6)	2.9 (4.7)	3.4 (2.4)	3.4 (2.0)	5.7 (3.3)	4.4 (2.3)	3.9 (3)	3.1 (2.9)
	Int/External Rotation	9.1 (5.4)	8.0 (3.1)	4.6 (1.2)	3.4 (0.8)	0.3 (0.4)	0.4 (0.4)	0.3 (0.4)	0.6 (0.4)	3.8 (4.4)	3.8 (4.5)	3.8 (4.7)	3.2 (3.7)	5.1 (4.3)	4.8 (4.3)	3.8 (3.1)	3.6 (3.7)
Knee right	Flexion/Extension	7.8 (4.7)	4.1 (2.8)	2.3 (1.1)	3.9 (1.5)	0.9 (0.1)	0.9 (0.1)	0.9 (0.1)	0.9 (0.0)	**3.9 (1.7)**	0.6 (1.6)	1.7 (1.5)	0.5 (0.9)	6.7 (4.6)	7.2 (4.1)	1.5 (3.9)	0.9 (3.8)
	Abd/Adduction	**7.4 (2.4)**	2.8 (1.8)	6.1 (2.4)	2.1 (2.4)	0.1 (0.4)	0.8 (0.5)	0.2 (0.4)	0.7 (0.5)	**7.7 (5.0)**	3.4 (4.4)	3.2 (4.2)	1.9 (2.1)	5.9 (4.6)	2.1 (2.6)	3.0 (2.4)	2.9 (2.4)
	Int/External Rotation	**8.4 (3.5)**	**6.3 (2.5)**	4.6 (4.7)	4.3 (2.0)	0.3 (0.5)	0.5 (0.5)	0.4 (0.4)	0.6 (0.2)	3.4 (3.5)	3.0 (2.5)	3.4 (1.9)	2.8 (1.8)	**7.5 (5.4)**	6.5 (3.2)	6.4 (3.3)	4.3 (2.5)
Ankle right	Dorsi/Plantarflexion	8.3 (4.1)	6.5 (4.4)	4.0 (1.8)	4.0 (1.7)	0.9 (0.1)	0.9 (0.1)	0.9 (0.1)	0.9 (0.1)	4.3 (4.8)	6.0 (4.8)	5.3 (4.2)	4.1 (2.9)	4.3 (4.4)	4.4 (4.1)	4.7 (2.3)	4.3 (2.3)
	Prono/Supination	9.2 (5.0)	6.7 (5.4)	6.2 (4.1)	5.4 (2.5)	0.5 (0.2)	0.6 (0.2)	0.6 (0.2)	0.6 (0.2)	8.0 (5.6)	2.7 (3.5)	3.7 (2.1)	3.2 (1.8)	5.5 (4.5)	4.3 (3.2)	4.3 (2.6)	4.3 (2.9)
Hip left	Flexion/Extension	7.5 (2.5)	5.2 (2.8)	4.1 (1.7)	3.6 (1.1)	0.9 (0.1)	0.9 (0.1)	0.9 (0.1)	0.9 (0.1)	2.9 (5.1)	4.4 (4.5)	3.8 (2.4)	4.1 (2.5)	5.8 (3.0)	5.9 (3.7)	5.8 (3.1)	5.4 (3.3)
	Abd/Adduction	5.1 (1.5)	3.7 (1.5)	4.1 (2.4)	3.5 (2.2)	0.6 (0.3)	0.8 (0.2)	0.8 (0.2)	0.8 (0.2)	4.4 (1.4)	3.5 (1.9)	3.8 (2.1)	4.1 (1.2)	7.3 (2.1)	7.6 (2.1)	4.8 (1.5)	4.0 (1.6)
	Int/External Rotation	**7.4 (4.4)**	7.0 (4.5)	4.9 (2.3)	3.7 (2.4)	0.2 (0.3)	0.5 (0.3)	0.2 (0.3)	0.5 (0.3)	4.3 (1.2)	5.2 (3.2)	5.4 (1.1)	3.4 (3.1)	**10.6 (2.6)**	8.2 (3.3)	6.1 (1.6)	4.6 (1.9)
Knee left	Flexion/Extension	5.9 (3.0)	3.9 (2.6)	2.6 (1.4)	2.5 (1.1)	0.9 (0.1)	0.9 (0.1)	0.9 (0.1)	0.9 (0.1)	2.7 (2.6)	0.9 (2.0)	1.4 (2.6)	1.4 (0.2)	**7.0 (3.0)**	5.7 (2.4)	2.4 (1.3)	2.3 (1.2)
	Abd/Adduction	**6.3 (2.4)**	3.2 (0.9)	5.3 (3.1)	2.9 (2.0)	0.4 (0.3)	0.4 (0.3)	0.4 (0.3)	0.6 (0.2)	5.4 (3.9)	2.8 (2.5)	6.1 (4.2)	2.5 (1.3)	10.6 (3.5)	3.7 (1.5)	6.9 (2.5)	4.2 (1.8)
	Int/External Rotation	7.6 (4.1)	5.8 (2.8)	7.1 (3.4)	4.7 (1.3)	0.2 (0.2)	0.3 (0.2)	0.2 (0.2)	0.5 (0.2)	5.2 (4.1)	3.9 (4.1)	4.2 (5.7)	3.0 (2.8)	5.8 (4.4)	7.5 (4.2)	6.2 (2.3)	4.4 (3.0)
Ankle left	Dorsi/Plantarflexion	5.1 (2.0)	4.5 (1.6)	3.1 (1.7)	3.1 (1.6)	0.9 (0.1)	0.9 (0.2)	0.9 (0.1)	0.9 (0.1)	3.5 (4.1)	6.6 (3.5)	4.4 (2.8)	3.5 (2.9)	4.5 (2.1)	3.5 (1.2)	4.4 (3.2)	3.9 (2.2)
	Prono/Supination	7.0 (4.1)	5.2 (2.5)	4.4 (3.5)	4.8 (1.4)	0.5 (0.2)	0.6 (0.2)	0.6 (0.2)	0.6 (0.2)	5.1 (2.8)	3.2 (6.4)	1.6 (1.6)	2.5 (1.2)	8.1 (4.0)	6.0 (3.8)	6.3 (2.7)	5.2 (2.5)

Table S2: Values between calibration methods and MoCap during ***walking*** *for joints-planes. RMSE: root mean square error, R^2: coefficient of determination, ΔROM: absolute difference in range of motion, ΔPeak: absolute difference in max peak, SD: standard deviation. Significant differences $p < 0.05$ – values in bold.*

Running		RMSE(°)				R^2				ΔROM(°)				Δpeak(°)			
		Method 0	Method 1	Method 2	Method 3	Method 0	Method 1	Method 2	Method 3	Method 0	Method 1	Method 2	Method 3	Method 0	Method 1	Method 2	Method 3
		Mean (SD)				Mean (SD)				Mean (SD)				Mean (SD)			
Pelvic	Tilt	5.4 (1.6)	6.7 (2.7)	3.5 (2.4)	3.8 (2.6)	0.5 (0.3)	0.5 (0.3)	0.6 (0.3)	0.6 (0.3)	2.4 (1.9)	2.2 (1.9)	2.4 (1.9)	2.2 (1.9)	12.1 (2.6)	10.3 (2.3)	9.7 (3.4)	4.6 (2.7)
	List	6.3 (1.9)	6.2 (1.0)	4.1 (1.4)	4.4 (1.0)	0.5 (0.3)	0.5 (0.3)	0.6 (0.3)	0.6 (0.3)	1.8 (1.9)	2.2 (1.9)	1.3 (1.7)	1.3 (1.8)	1.1 (0.8)	1.4 (0.8)	2.2 (0.8)	1.7 (0.7)
	Rotation	**16 (5.4)**	6.7 (2.9)	4.1 (1.3)	4.0 (0.6)	0.3 (0.2)	0.5 (0.3)	0.6 (0.2)	0.4 (0.2)	3.8 (1.4)	4.7 (1.1)	3.4 (1.0)	4.0 (1.0)	**16.0 (4.6)**	2.9 (2.9)	3.2 (3.2)	2.5 (3.2)
Lumbar	Extension	7.4 (2.4)	4.6 (3.7)	5.6 (2.4)	4.9 (2.5)	0.8 (0.2)	0.8 (0.1)	0.8 (0.1)	0.8 (0.1)	3.5 (1.7)	2.2 (1.9)	1.8 (1.9)	2.3 (1.8)	10.3 (3.3)	6.7 (2.2)	5.3 (3.9)	4.8 (2.7)
	Bending	4.8 (1.2)	4.0 (1.1)	4.8 (1.2)	4.4 (1.3)	0.5 (0.3)	0.6 (0.2)	0.7 (0.2)	0.7 (0.2)	5.1 (3.5)	3.3 (3.7)	3.8 (3.5)	4.0 (3.5)	6.5 (1.8)	4.2 (1.9)	5.6 (2.4)	5.5 (2.0)
	Rotation	4.8 (1.2)	5.1 (1.6)	5.8 (0.6)	4.7 (1.2)	0.9 (0.3)	0.9 (0.1)	0.9 (0.1)	0.9 (0.1)	4.4 (2.2)	4.5 (2.5)	4.5 (2.6)	4.5 (2.5)	8.1 (2.3)	6.3 (2.0)	4.5 (2.1)	5.3 (2.1)
Hip right	Flexion/Extension	7.9 (5.9)	4.4 (3.5)	4.4 (2.0)	5.1 (1.8)	0.8 (0.4)	0.9 (0.1)	0.8 (0.4)	0.8 (0.4)	2.5 (0.8)	2.7 (0.7)	2.4 (0.8)	1.2 (0.5)	5.0 (2.3)	6.8 (2.9)	6.4 (3.5)	5.8 (2.9)
	Abd/Adduction	7.2 (5.2)	4.4 (2.5)	3.9 (0.6)	2.4 (1.6)	0.5 (0.5)	0.8 (0.5)	0.6 (0.4)	0.9 (0.2)	6.6 (1.5)	1.6 (1.6)	3.5 (1.6)	3.5 (1.6)	7.0 (2.4)	4.2 (1.6)	4.9 (1.3)	4.5 (1.5)
	Int/External Rotation	9.1 (5.0)	5.1 (1.7)	5.2 (1.7)	4.0 (1.7)	0.2 (0.5)	0.2 (0.4)	0.5 (0.5)	0.6 (0.5)	4.5 (1.5)	5.1 (1.4)	4.9 (1.4)	4.6 (1.7)	5.0 (1.6)	5.5 (1.7)	5.2 (1.1)	4.1 (1.5)
Knee right	Flexion/Extension	**9.8 (4.0)**	4.2 (1.9)	4.6 (2.0)	3.5 (3.0)	0.7 (0.6)	0.9 (0.1)	0.7 (0.6)	0.9 (0.1)	4.8 (2.1)	4.7 (1.9)	5.1 (1.7)	4.9 (2.1)	6.6 (2.3)	5.1 (1.9)	3.2 (1.2)	2.9 (0.8)
	Abd/Adduction	**8.7 (4.5)**	4.7 (3.3)	6.0 (4.9)	5.6 (2.7)	0.2(0.6)	0.8 (0.5)	0.3 (0.5)	0.7 (0.5)	5.4 (3.3)	1.5 (1.0)	3.6 (1.0)	2.9 (1.2)	7.6 (3.6)	2.0 (1.4)	4.2 (1.1)	3.8 (1.4)
	Int/External Rotation	9.5 (4.9)	7.1 (6.0)	6.7 (5.0)	4.8 (3.1)	0.3 (0.6)	0.4 (0.5)	0.3 (0.6)	0.4 (0.5)	6.3 (1.2)	5.7 (1.8)	4.8 (1.8)	3.5 (3.0)	5.1 (2.7)	6.8 (3.2)	6.2 (3.0)	3.5 (2.2)
Ankle right	Dorsi/Plantarflexion	8.6 (5.9)	4.2 (3.3)	3.4 (2.1)	4.2(2.5)	0.7 (0.6)	0.9 (0.1)	0.7 (0.6)	0.9 (0.1)	7.2 (3.1)	4.0 (2.5)	4.1 (1.2)	4.5 (2.1)	4.4 (2.4)	4.7 (2.8)	4.1 (1.9)	5.2 (1.9)
	Prono/Supination	10.5 (2.4)	9.2 (1.5)	5.1 (1.5)	5.2 (1.1)	0.6 (0.1)	0.8 (0.1)	0.6 (0.2)	0.7 (0.3)	8.3 (3.2)	2.5 (1.2)	5.5 (1.2)	3.1 (1.7)	5.1 (1.5)	3.5 (1.5)	3.0 (1.2)	3.7 (1.2)
Hip left	Flexion/Extension	6.8 (2.4)	11.2 (2.7)	4.5 (3.8)	4.6 (2.7)	0.9 (0.1)	0.9 (0.1)	0.9 (0.1)	0.9 (0.1)	2.9 (0.9)	2.6 (1.1)	2.9 (1.0)	3.0 (1.1)	5.1 (3.4)	8.3 (3.1)	4.9 (2.3)	4.2 (2.2)
	Abd/Adduction	5.9 (1.3)	5.6 (0.6)	6.4 (1.3)	4.2 (1.2)	0.2 (0.3)	0.7 (0.3)	0.8 (0.3)	0.8 (0.3)	2.3 (1.4)	2.3 (1.7)	2.3 (1.8)	2.4 (1.8)	5.5 (1.6)	3.5 (1.2)	6.1 (1.8)	4.6 (1.8)
	Int/External Rotation	7.8 (2.5)	7.4 (2.1)	5.5 (2.5)	5.0 (1.8)	0.2 (0.2)	0.2 (0.2)	0.4 (0.2)	0.5 (0.2)	4.8 (2.2)	4.4 (2.2)	4.4 (3.1)	4.0 (3.0)	6.6 (2.4)	5.4 (2.6)	7.8 (2.5)	5.1 (2.7)
Knee left	Flexion/Extension	6.9 (2.1)	6.3 (1.7)	6.6 (2.4)	4.9 (1.8)	0.9 (0.1)	0.9 (0.1)	0.9 (0.1)	0.9 (0.1)	4.9 (3.0)	3.0 (2.2)	5.2 (3.0)	3.7 (2.2)	6.3 (1.9)	6.2 (1.7)	4.4 (1.1)	3.5 (1.1)
	Abd/Adduction	**9.3 (2.1)**	6.2 (1.0)	4.2 (1.2)	4.1 (1.7)	0.4 (0.3)	0.3 (0.3)	0.5 (0.3)	0.5 (0.2)	9.2 (4.3)	5.8 (2.8)	4.2 (3.1)	4.1 (1.2)	**5.1 (1.2)**	1.6 (2.1)	4.1 (2.1)	2.1 (1.5)
	Int/External Rotation	8.2 (2.3)	6.3 (2.5)	6.0 (2.7)	4.8 (1.5)	0.3 (0.3)	0.4 (0.2)	0.3 (0.3)	0.5 (0.2)	5.4 (3.5)	4.8 (2.9)	6.3 (3.3)	4.7 (1.6)	5.4 (2.7)	6.1 (3.2)	7.2 (4.0)	3.5 (2.4)
Ankle left	Dorsi/Plantarflexion	4.9 (1.5)	4.9 (0.9)	4.6 (1.4)	4.8 (1.4)	0.9 (0.1)	0.9 (0.1)	0.9 (0.1)	0.9 (0.1)	9.3 (2.6)	7.9 (3.4)	7.9 (2.8)	5.4 (2.7)	7.1 (3.0)	6.1 (3.1)	4.4 (1.8)	4.0 (2.0)
	Prono/Supination	8.2 (2.1)	5.8 (2.0)	8.8 (2.5)	6.1 (1.9)	0.4 (0.3)	0.4 (0.3)	0.3 (0.3)	0.5 (0.2)	4.8 (0.9)	5.9 (1.7)	4.0 (3.2)	2.9 (2.6)	6.9 (2.6)	4.3 (2.6)	9.3 (2.2)	5.0 (1.8)

*Table S3: Values between calibration methods and MoCap during **running** for joints-planes. RMSE: root mean square error, R^2: coefficient of determination, ΔROM: absolute difference in range of motion, ΔPeak: absolute difference in max peak, SD: standard deviation. Significant differences $p < 0.05$ – values in bold.*

Stairs ascent		RMSE(°)				R^2				ΔROM(°)				Δpeak(°)			
		Method 0	Method 1	Method 2	Method 3	Method 0	Method 1	Method 2	Method 3	Method 0	Method 1	Method 2	Method 3	Method 0	Method 1	Method 2	Method 3
		Mean (SD)				Mean (SD)				Mean (SD)				Mean (SD)			
Pelvic	Tilt	8.4 (2.5)	8.4 (3.3)	7.3 (2.7)	7.2 (3.1)	0.7 (0.3)	0.7 (0.3)	0.7 (0.3)	0.7 (0.3)	2.7 (0.8)	2.7 (0.9)	2.1 (0.9)	2.1 (0.9)	12.1 (2.6)	10.3 (2.3)	9.7 (3.4)	4.6 (2.7)
	List	9.3 (2.7)	8.8 (2.7)	8.0 (2.9)	6.8 (2.9)	0.9 (0.3)	0.8 (0.3)	0.9 (0.3)	0.9 (0.3)	4.8 (1.9)	4.2 (1.9)	4.3 (1.7)	4.3 (1.8)	5.1 (0.8)	5.4 (0.8)	5.2 (0.8)	5.7 (0.7)
	Rotation	5.5 (3.0)	6.8 (2.6)	5.6 (3.0)	5.6 (3.0)	0.4 (0.3)	0.4 (0.3)	0.4 (0.3)	0.5 (0.3)	4.8 (1.4)	5.7 (1.6)	4.4 (1.5)	5.0 (1.0)	14.0 (4.6)	8.9 (2.4)	5.2 (3.2)	4.5 (1.2)
Lumbar	Extension	6.9 (3.0)	11 (4.7)	4.2 (3.4)	4.0 (3.4)	0.4 (0.2)	0.6 (0.2)	0.6 (0.2)	0.5 (0.2)	2.5 (0.7)	2.1 (0.7)	2.4 (0.7)	1.9 (0.8)	10.4 (3.3)	4.8 (3.2)	8.1 (3.8)	3.2 (3.8)
	Bending	5.5 (1.7)	3.9 (1.2)	4.7 (1.2)	4.3 (1.2)	0.4 (0.4)	0.7 (0.3)	0.8 (0.3)	0.8 (0.3)	5.6 (3.8)	5.0 (3.6)	3.0 (3.5)	4.2 (3.6)	3.5 (3.1)	3.6 (2.9)	4.4 (2.5)	5.0 (3.0)
	Rotation	9.5 (3.2)	10 (3.3)	3.5 (3.3)	3.4 (3.4)	0.7 (0.3)	0.7 (0.3)	0.8 (0.3)	0.8 (0.3)	2.8 (1.8)	2.1 (1.8)	2.3 (1.6)	2.5 (1.5)	2.7 (2.0)	4.3 (2.4)	2.9 (2.5)	3.3 (2.5)
Hip right	Flexion/Extension	7.9 (4.9)	5.4 (2.5)	2.4 (3.0)	2.1 (1.8)	0.9 (0.1)	0.9 (0.1)	0.9 (0.1)	0.9 (0.1)	3.5 (1.8)	2.7 (1.7)	3.4 (1.8)	3.2 (1.5)	7.0 (3.3)	5.6 (3.9)	4.1 (3.5)	4.8 (1.9)
	Abd/Adduction	**9.2 (4.2)**	**9.8 (2.4)**	6.1 (1.6)	2.4 (1.9)	0.5 (0.5)	0.8 (0.2)	0.8 (0.2)	0.8 (0.2)	5.6 (2.5)	3.6 (1.6)	2.5 (2.6)	4.5 (1.6)	7.6 (4.4)	2.7 (1.9)	3.5 (2.3)	3.4 (2.3)
	Int/External Rotation	9.7 (3.0)	4.1 (3.7)	2.2 (3.7)	4.0 (2.7)	0.4 (0.5)	0.7 (0.3)	0.4 (0.4)	0.7 (0.3)	4.5 (1.4)	5.1 (1.3)	4.7 (1.8)	4.8 (1.8)	5.2 (1.8)	5.2 (1.8)	5.5 (1.8)	4.5 (1.7)
Knee right	Flexion/Extension	7.8 (5.0)	4.2 (2.9)	4.6 (3.0)	3.5 (2.0)	0.9 (0.1)	0.9 (0.1)	0.9 (0.1)	0.9 (0.1)	7.0 (2.1)	5.5 (2.6)	5.3 (2.5)	5.1 (1.8)	6.6 (2.3)	5.1 (1.9)	3.2 (1.2)	2.9 (0.8)
	Abd/Adduction	**9.7 (4.5)**	4.4 (2.3)	4.0 (2.4)	3.6 (2.1)	0.2 (0.5)	0.3 (0.3)	0.2 (0.5)	0.5 (0.3)	6.4 (3.3)	5.5 (1.0)	6.6 (1.0)	5.9 (1.2)	**7.6 (3.6)**	2.0 (1.4)	4.2 (1.1)	3.8 (1.4)
	Int/External Rotation	9.0 (3.9)	5.1 (3.0)	6.2 (2.0)	4.8 (3.1)	0.6 (0.4)	0.2 (0.3)	0.4 (0.4)	0.6 (0.3)	5.3 (1.2)	5.0 (1.8)	2.8 (1.8)	2.5 (1.4)	9.1 (2.7)	5.8 (3.2)	9.4 (3.0)	4.5 (2.2)
Ankle right	Dorsi/Plantarflexion	6.6 (2.9)	6.2 (3.3)	4.4 (4.1)	4.2(2.5)	0.9 (0.1)	0.9 (0.1)	0.9 (0.1)	0.9 (0.1)	7.4 (3.1)	4.0 (2.5)	4.1 (1.2)	4.3 (2.5)	4.9 (2.5)	4.3 (2.2)	5.1 (1.2)	4.8 (1.5)
	Prono/Supination	10.5 (2.4)	9.2 (1.5)	5.1 (1.5)	5.2 (1.1)	0.6 (0.1)	0.8 (0.1)	0.6 (0.2)	0.7 (0.3)	8.2 (3.2)	2.7 (1.2)	2.5 (1.2)	2.3 (1.7)	5.0 (1.0)	3.5 (1.8)	4.0 (1.8)	3.9 (1.9)
Hip left	Flexion/Extension	5.5 (2.7)	4.6 (3.5)	4.6 (3.2)	4.6 (2.7)	0.9 (0.1)	0.9 (0.1)	0.9 (0.1)	0.9 (0.1)	2.9 (0.9)	2.6 (1.1)	2.9 (1.0)	3.0 (1.1)	5.1 (3.4)	8.3 (3.1)	4.9 (2.3)	4.2 (2.2)
	Abd/Adduction	7.2 (1.3)	4.8 (0.6)	7.1 (1.8)	4.2 (1.8)	0.8 (0.3)	0.8 (0.3)	0.7 (0.4)	0.8 (0.3)	2.3 (1.4)	2.3 (1.7)	2.3 (1.8)	2.4 (1.8)	5.5 (1.6)	3.5 (1.2)	6.1 (1.8)	4.6 (1.8)
	Int/External Rotation	**9.3 (2.2)**	4.5 (2.2)	5.4 (3.1)	5.0 (1.5)	6.6 (2.4)	5.4 (2.6)	7.8 (2.5)	5.1 (2.7)	4.6 (1.2)	4.6 (1.3)	4.0 (1.8)	4.0 (1.6)	5.5 (1.2)	4.5 (1.9)	6.5 (1.8)	4.5 (1.9)
Knee left	Flexion/Extension	7.3 (2.1)	6.4 (1.3)	6.9 (2.0)	5.0 (1.6)	0.9 (0.1)	0.9 (0.1)	0.9 (0.1)	0.9 (0.1)	4.3 (4.0)	5.0 (2.6)	3.9 (2.0)	4.7 (2.1)	7.3 (1.2)	9.2 (1.5)	5.1 (1.5)	5.2 (1.9)
	Abd/Adduction	**11.3 (1.1)**	4.2 (1.6)	6.5 (1.2)	4.5 (1.8)	0.4 (0.3)	0.4 (0.3)	0.4 (0.3)	0.5 (0.3)	10.5 (1.3)	5.4 (2.9)	3.5 (2.1)	5.1 (1.2)	7.1 (1.2)	4.6 (2.1)	8.1 (2.1)	4.1 (1.5)
	Int/External Rotation	**11.6 (2.3)**	7.3 (1.5)	5.8 (2.9)	4.9 (1.9)	0.1 (0.3)	0.4 (0.3)	0.4 (0.3)	0.5 (0.3)	5.5 (3.0)	3.7 (1.5)	5.7 (2.2)	5.7 (2.2)	5.7 (2.2)	7.7 (3.2)	7.5 (3.0)	4.2 (2.4)
Ankle left	Dorsi/Plantarflexion	6.9 (1.5)	5.2 (0.9)	4.6 (2.4)	4.8 (1.9)	0.9 (0.1)	0.9 (0.2)	0.9 (0.1)	0.9 (0.1)	7.2 (2.6)	4.8 (3.4)	6.3 (2.8)	5.6 (2.7)	6.1 (3.0)	4.9 (3.1)	4.9 (1.8)	4.8 (2.0)
	Prono/Supination	8.2 (2.1)	5.8 (2.0)	8.8 (2.5)	6.1 (1.9)	0.2 (0.3)	0.2 (0.4)	0.3 (0.1)	0.5 (0.3)	5.8 (0.9)	5.4 (1.7)	5.6 (3.2)	4.9 (2.6)	6.9 (2.6)	4.8 (2.6)	7.7 (2.2)	6.0 (1.2)

*Table S4: Values between calibration methods and MoCap during **stairs ascent** for joints-planes. RMSE: root mean square error, R^2: coefficient of determination, ΔROM: absolute difference in range of motion, ΔPeak: absolute difference in max peak, SD: standard deviation. Significant differences $p < 0.05$ – values in bold.*

Stairs descent		RMSE(°)				R^2				ΔROM(°)				Δpeak(°)			
		Method 0	Method 1	Method 2	Method 3	Method 0	Method 1	Method 2	Method 3	Method 0	Method 1	Method 2	Method 3	Method 0	Method 1	Method 2	Method 3
		Mean (SD)				Mean (SD)				Mean (SD)				Mean (SD)			
Pelvic	Tilt	11 (4.6)	9.8 (3.5)	5.9 (7.1)	5.9 (5.2)	0.5 (0.2)	0.5 (0.1)	0.6 (0.2)	0.6 (0.1)	2.0 (0.8)	1.8 (0.9)	1.2 (1.9)	1.2 (0.9)	**14.1 (2.6)**	12.3 (2.3)	10.7 (3.4)	8.6 (2.7)
	List	4.4 (1.5)	3.8 (1.4)	2.9 (1.5)	2.5 (1.5)	0.8 (0.5)	0.7 (0.5)	0.8 (0.4)	0.8 (0.2)	1.3 (1.1)	1.8 (1.1)	1.5 (1.7)	1.8 (1.0)	3.6 (0.8)	2.3 (0.8)	2.7 (0.8)	2.0 (0.7)
	Rotation	9.1 (2.9)	7.4 (3.0)	4.0 (3.0)	4.0 (3.0)	0.8 (0.5)	0.8 (0.3)	0.8 (0.3)	0.8 (0.3)	4.8 (1.4)	5.7 (1.4)	4.4 (1.5)	5.0 (1.0)	10.0 (4.6)	6.9 (2.4)	9.2 (3.2)	5.2 (1.2)
Lumbar	Extension	6.8 (3.0)	10 (4.1)	4.4 (3.1)	4.4 (2.8)	0.7 (0.1)	0.8 (0.1)	0.7 (0.3)	0.8 (0.2)	4.0 (0.7)	2.6 (0.5)	3.2 (1.7)	3.0 (1.8)	8.4 (3.3)	8.2 (3.2)	4.4 (3.8)	4.2 (3.8)
	Bending	4.1 (1.3)	2.7 (0.7)	2.2 (1.1)	2.7 (0.9)	0.7 (0.3)	0.9 (0.3)	0.9 (0.3)	0.9 (0.2)	3.3 (1.4)	3.3 (1.1)	3.2 (1.5)	3.5 (1.1)	1.6 (1.1)	1.6 (1.9)	2.4 (1.5)	2.0 (0.6)
	Rotation	9.9 (3.2)	10 (4.1)	3.7 (3.3)	3.4 (3.4)	0.9 (0.4)	0.9 (0.3)	0.9 (0.2)	0.9 (0.2)	4.7 (1.4)	4.9 (1.8)	5.2 (1.4)	5.1 (1.5)	3.7 (1.8)	3.3 (2.4)	5.9 (2.5)	2.2 (2.5)
Hip right	Flexion/Extension	**12 (5.0)**	**11 (4.6)**	4.4 (4.0)	4.4 (3.5)	0.9 (0.1)	0.9 (0.1)	0.9 (0.1)	0.9 (0.1)	3.2 (1.4)	2.9 (1.7)	3.1 (1.8)	3.1 (1.4)	8.5 (3.3)	9.3 (3.92	4.1 (3.5)	3.8 (1.9)
	Abd/Adduction	**7.2 (2.1)**	**7.4 (2.5)**	3.9 (2.5)	2.4 (3.0)	0.7 (0.3)	0.8 (0.3)	8.0 (0.2)	0.8 (0.1)	3.6 (1.5)	2.8 (1.6)	2.4 (1.6)	2.4 (1.6)	7.7 (4.4)	4.2 (1.9)	3.4 (2.3)	3.2 (2.3)
	Int/External Rotation	**9.1 (5.0)**	8.1 (2.7)	6.2 (4.7)	4.0 (1.7)	0.6 (0.3)	0.6 (0.4)	0.7 (0.4)	0.7 (0.3)	4.9 (1.4)	4.2 (2.3)	5.6 (1.4)	5.5 (1.3)	6.3 (1.8)	7.5 (1.4)	4.8 (1.8)	5.5 (1.7)
Knee right	Flexion/Extension	**12 (5.2)**	13 (4.9)	4.6 (1.6)	3.5 (3.1)	0.9 (0.1)	0.9 (0.1)	0.9 (0.1)	0.9 (0.1)	6.9 (2.1)	3.5 (2.4)	3.1 (2.5)	3.2 (1.8)	7.2 (2.3)	4.7 (1.92	2.3 (1.2)	2.4 (0.8)
	Abd/Adduction	**8.7 (4.0)**	4.2 (2.8)	4.0 (1.9)	4.6 (2.6)	0.3 (0.3)	0.4 (0.3)	0.5 (0.3)	0.5 (0.2)	4.2 (3.3)	3.6 (1.3)	2.1 (1.7)	1.9 (2.7)	6.5 (2.6)	3.8 (1.4)	4.0 (1.1)	3.6 (1.4)
	Int/External Rotation	**7.5 (2.1)**	5.3 (2.0)	8.6 (2.4)	5.1 (2.5)	0.2 (0.3)	0.6 (0.3)	0.4 (0.3)	0.6 (0.3)	4.6 (1.5)	3.3 (1.8)	3.6 (1.4)	2.3 (2.4)	8.8 (2.7)	5.2 (2.2)	9.0 (3.0)	6.1 (2.1)
Ankle right	Dorsi/Plantarflexion	12 (3.2)	12 (3.6)	3.4 (2.1)	4.2(2.6)	0.9 (0.1)	0.9 (0.1)	0.9 (0.1)	0.9 (0.1)	7.3 (3.1)	3.8 (2.5)	5.1 (1.2)	5.3 (1.5)	6.3 (2.5)	6.3 (2.2)	5.3 (1.4)	5.3 (1.5)
	Prono/Supination	10.5 (2.4)	9.2 (1.5)	5.1 (1.5)	5.2 (1.1)	0.6 (0.1)	0.8 (0.1)	0.6 (0.2)	0.7 (0.3)	8.2 (3.2)	5.7 (1.4)	5.5 (1.4)	5.3 (1.7)	12.0 (1.0)	8.5 (1.8)	12.0 (1.4)	7.2 (1.9)
Hip left	Flexion/Extension	**7.3 (2.1)**	**9.4 (1.3)**	4.7 (2.0)	5.1 (1.6)	0.9 (0.1)	0.9 (0.1)	0.9 (0.1)	0.9 (0.1)	3.0 (0.9)	2.4 (1.1)	2.9 (1.0)	2.5 (1.1)	4.6 (3.4)	8.7 (3.4)	2.4 (2.3)	2.8 (2.1)
	Abd/Adduction	4.9 (1.3)	3.5 (0.6)	5.1 (1.8)	3.7 (1.8)	0.4 (0.3)	0.7 (0.3)	0.8 (0.3)	0.7 (0.2)	3.0 (1.4)	2.8 (1.7)	3.0 (1.8)	3.0 (0.9)	4.2 (1.6)	2.4 (1.2)	5.3 (1.8)	3.1 (1.8)
	Int/External Rotation	6.6 (2.2)	5.5 (2.2)	5.4 (3.1)	6.1 (1.5)	0.5 (0.3)	0.5 (0.4)	0.6 (0.3)	0.5 (0.3)	5.0 (2.2)	5.5 (2.3)	5.1 (2.8)	5.0 (2.8)	7.4 (4.2)	5.4 (1.9)	7.1 (1.8)	5.5 (1.9)
Knee left	Flexion/Extension	8.4 (2.2)	6.3 (2.1)	6.4 (2.5)	4.4 (2.4)	0.9 (0.1)	0.9 (0.1)	0.9 (0.1)	0.9 (0.1)	3.6 (4.0)	3.9 (2.6)	2.4 (2.0)	2.6 (2.1)	6.6 (2.7)	7.6 (2.7)	4.1 (1.4)	3.8 (3.4)
	Abd/Adduction	**9.6 (1.9)**	5.3 (0.6)	8.7 (1.8)	4.7 (2.2)	0.5 (0.3)	0.2 (0.3)	0.2 (0.3)	0.4 (0.3)	6.8 (2.3)	1.7 (2.9)	4.5 (2.1)	5.2 (1.2)	11.1 (3.2)	6.3 (2.1)	5.5 (3.1)	5.1 (2.0)
	Int/External Rotation	11.2 (2.2)	8.2 (1.2)	11.1 (2.9)	5.9 (2.9)	0.1 (0.3)	0.6 (0.3)	0.1 (0.2)	0.4 (0.2)	7.7 (3.0)	5.5 (2.4)	5.1 (2.0)	4.9 (2.0)	6.4 (2.2)	6.7 (3.2)	7.6 (3.0)	6.2 (2.4)
Ankle left	Dorsi/Plantarflexion	7.4 (1.5)	6.4 (1.0)	6.0 (1.4)	6.1 (1.1)	0.9 (0.1)	0.9 (0.2)	0.9 (0.1)	0.9 (0.1)	8.4 (2.6)	7.3 (2.4)	6.1 (2.0)	5.2 (2.0)	5.5 (2.0)	4.5 (2.4)	3.1 (1.8)	3.1 (2.0)
	Prono/Supination	9.3 (2.1)	6.9 (2.0)	9.0 (2.5)	7.2 (1.9)	0.2 (0.3)	0.4 (0.4)	0.2 (0.1)	0.4 (0.3)	6.4 (0.9)	2.0 (2.7)	6.0 (3.2)	3.9 (2.5)	6.5 (2.6)	4.4 (2.6)	7.2 (2.2)	5.8 (1.6)

Table S5: Values between calibration methods and MoCap during stairs descent for joints-planes. RMSE: root mean square error, R^2: coefficient of determination, ΔROM: absolute difference in range of motion, ΔPeak: absolute difference in max peak, SD: standard deviation. Significant differences $p < 0.05$ – values in bold.
